# Efficacy and Safety of Low-Intensity Pulsed Ultrasound-Induced Blood–Retinal Barrier Opening in Mice

**DOI:** 10.3390/pharmaceutics15071896

**Published:** 2023-07-06

**Authors:** Alexandre Bourdin, Manon Ortoli, Remi Karadayi, Lauriane Przegralek, Florian Sennlaub, Bahram Bodaghi, Xavier Guillonneau, Alexandre Carpentier, Sara Touhami

**Affiliations:** 1 Institut de la Vision, Sorbonne Université, INSERM, CNRS, 75012 Paris, France; alexandre.bourdin@ens.uvsq.fr (A.B.); manon.ortoli@hotmail.fr (M.O.); remi.karadayi@inserm.fr (R.K.); lauriane.przegralek@inserm.fr (L.P.); florian.sennlaub@inserm.fr (F.S.); xavier.guillonneau@inserm.fr (X.G.); 2 Ophthalmology Department, Pitié Salpêtrière University Hospital, AP-HP, Sorbonne Université, 75013 Paris, France; bahram.bodaghi@aphp.fr; 3 Department of Neurosurgery, Pitié Salpêtrière University Hospital, AP-HP, Sorbonne Université, 75013 Paris, France; alexandre.carpentier@aphp.fr; 4 NeurOn Brain Machine Interface Clinical Research Group, Pitié Salpêtrière University Hospital, AP-HP, Sorbonne Université, 75013 Paris, France; 5 ASTRL Advanced Surgical Technologies Research Laboratory, Sorbonne Université, 75013 Paris, France

**Keywords:** sonication, sonoporation, sonopermeation, microbubbles, retina, ultrasound, blood–retina barrier

## Abstract

Systemic drugs can treat various retinal pathologies such as retinal cancers; however, their ocular diffusion may be limited by the blood–retina barrier (BRB). Sonication corresponds to the use of ultrasound (US) to increase the permeability of cell barriers including in the BRB. The objective was to study the efficacy and safety of sonication using microbubble-assisted low-intensity pulsed US in inducing a transient opening of the BRB. The eyes of C57/BL6J mice were sonicated at different acoustic pressures (0.10 to 0.50 MPa). Efficacy analyses consisted of fluorescein angiography (FA) performed at different timepoints and the size of the leaked molecules was assessed using FITC-marked dextrans. Tolerance was assessed by fundus photographs, optical coherence tomography, immunohistochemistry, RT-qPCR, and electroretinograms. Sonication at 0.15 MPa was the most suitable pressure for transient BRB permeabilization without altering the morphology or function of the retina. It did not increase the expression of inflammation or apoptosis markers in the retina, retinal pigment epithelium, or choroid. The dextran assay suggested that drugs up to 150 kDa in size can cross the BRB. Microbubble-assisted sonication at an optimized acoustic pressure of 0.15 MPa provides a non-invasive method to transiently open the BRB, increasing the retinal diffusion of systemic drugs without inducing any noticeable side-effect.

## 1. Introduction

Several strategies are used for drug delivery in ophthalmology. While the topical route is commonly used for ocular surface and anterior segment pathology, periocular and intraocular injections are preferred for diseases affecting the posterior segment, allowing the appropriate drug concentrations to reach the retina, retinal pigment epithelium (RPE), and choroid. Intravitreal injections are widely used in diseases including diabetic retinopathy, age related macular degeneration, inflammatory conditions, or cancers. However, they come with a real burden for patients and physicians because of their potential complications (increased ocular pressure, infection, retinal detachment, etc.) and the necessity of repeated injections over months, if not years. Systemic treatments can also be used in eye care, for example, in severe or bilateral ocular inflammation, particularly when associated with systemic manifestations, or in cancers such as in the case of retinoblastoma. The main challenge of systemic treatments is their intraocular diffusion, which can be limited by the blood–retinal barrier (BRB). Therefore, high doses are often required to reach sufficient retinal concentrations, which can cause systemic side effects. One solution to improve the efficacity of systemic drugs and reduce their iatrogenicity would be to temporarily open the BRB in order to enhance their diffusion.

The outer BRB is formed by the RPE, whereas the inner BRB isolates the retina from blood vessels through tight and adherens junctions between endothelial cells supported by pericytes and glial cells (astrocytes, Müller cells, and microglia) [1]. Tight junctions are composed of over 40 transmembrane and intracellular proteins including occludin, claudin, and zonula occludens (ZO) family proteins. In contrast, adherens junctions include vascular endothelial (VE)-cadherins complexed with β-catenins and α-catenins [1].

Drug delivery can be enhanced by sonication, using low-intensity pulsed ultrasound (US) waves combined with the intravascular injection of microbubbles [2]. Sonication induces microbubble oscillation and explosion, and thereby pore formation, endocytosis, and cell–cell junction opening. This mechanical stress enhances the extravasation of co-administered drugs. This strategy has shown to safely and transiently open the blood–brain barrier in animal models [3], improving drug distribution [4]. Sonication has been used in preclinical [5] and clinical [6,7] trials for the treatment of glioblastoma, allowing for the survival rates to be prolonged, at the cost of transient adverse events. Unlike internal organs, the eye is easily accessible to pulsed ultrasound-based treatments, making this technology suitable for future use in humans. In ophthalmology, the opening of the BRB by sonication has been investigated in a few studies. Hirokawa et al. first investigated the effects of sonication in rabbit eyes, showing poor leakage results on fluorescein angiography assays [8]. Park et al. investigated the effect of sonication at different pressure amplitudes in rats [9], showing transient BRB disruption without any distinguishable histological change, except for the presence of petechiae at the highest pressures. However, their study used a magnetic resonance imaging (MRI) contrast agent to visualize the opening of the BRB, which does not correlate precisely with the drugs that are routinely used for the management of patients in ophthalmology. In addition, the authors did not investigate the safety issues at a cellular, molecular, and functional level, which is of paramount importance when considering future clinical trials. More recently, Touahri et al. demonstrated the use of focused ultrasound associated with microbubbles to open the inner BRB in rats and allow the passage of adeno-associated viruses and macromolecules to the retina [10]. Rousou et al. also showed the passage of dextrans up to 20 kDa in ex vivo porcine eyes using 2 min-microbubble-assisted sonication at two peak-negative pressures of 0.3 and 0.6 MPa [11].

In this study, we investigated the efficacy and safety of low-intensity pulsed ultrasound sonication associated with the injection of microbubbles in mice. First, BRB opening and optimal acoustic parameters were assessed with fluorescein angiography. The degree of BRB opening was then quantified using various sizes of fluorescent dextrans. We also studied safety issues more precisely than in previous studies, with immunohistochemistry, gene expression, and electrophysiology to assess the visual function.

## 2. Materials and Methods

### 2.1. Animals

Wild-type (WT) C57BL/6J male mice, aged 8 to 12 weeks were obtained from Janvier Labs. The mice were kept to the indicated ages under specific pathogen-free conditions in a 12 h/12 h light/dark (100 lx) cycle with no additional cover in the cage and with water and normal diet available *ad libitum*. All experimental protocols and procedures were approved by the French Ministry of Higher Education, Research, and Innovation (authorization number APAFIS#27643). All procedures were performed under anesthesia and all efforts were made to minimize their suffering.

### 2.2. Ultrasound Sonication

**Figure 1:**
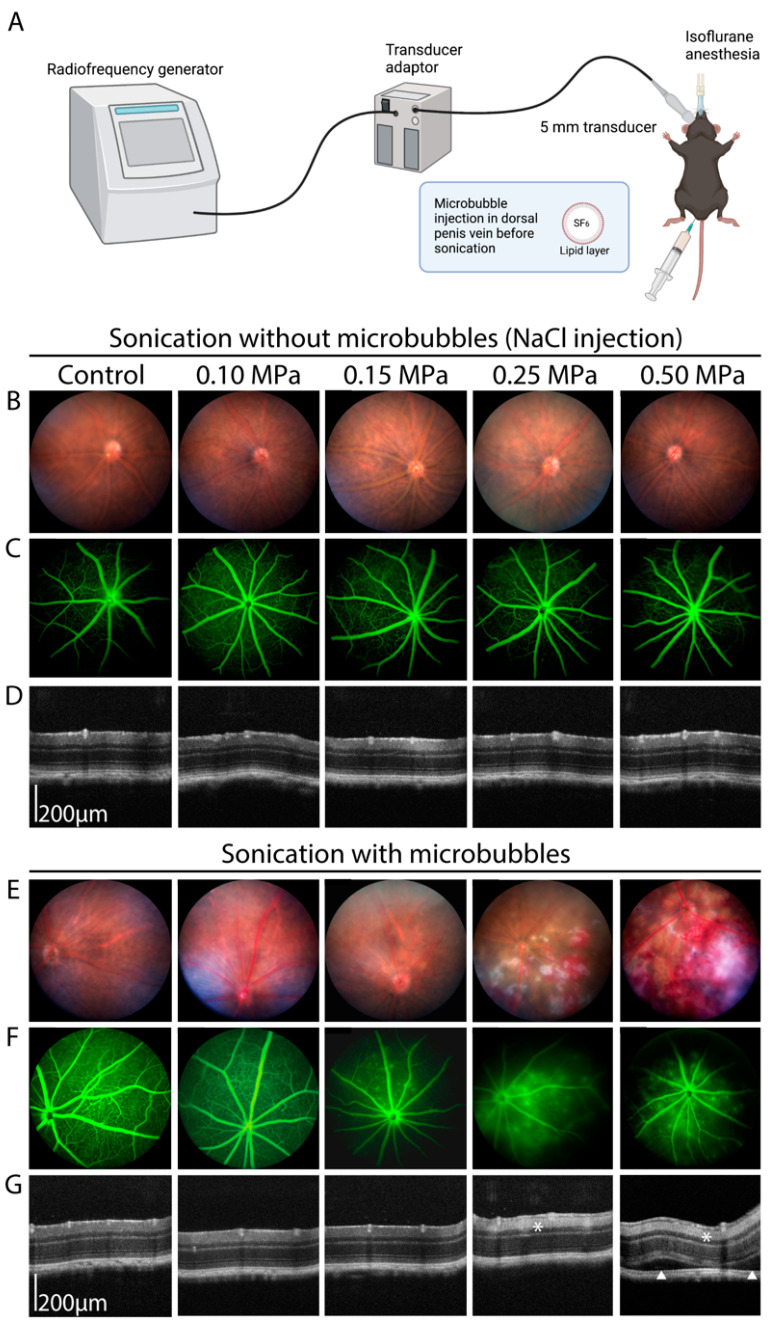
(**A**) Schematic representation of the sonication process, created with BioRender.com. (**B**–**G**) Multimodal imaging of C57/BL6J male mice undergoing low-intensity pulsed ultrasound sonication using different acoustic pressures, without (**B**–**D**) or with (**E**–**G**) microbubble injection. (**B**,**E**) Fundus retinophotograph, field of view 50°. (**C**,**F**) Fluorescein angiography (5 min, late phase), field of view 50°. (**D**,**G**) Optical coherence tomography. The white asterisks show a hyperreflectivity of the inner retinal layers, witnessing an ischemic process. The white arrowheads show subretinal detachments (n = 5–8/group).

For each experiment, the right eye of each mouse was sonicated using a 5 mm transducer driven by a custom radiofrequency generator (CarThera, Paris, France) [12] comprising a signal generator, a radiofrequency amplifier, an electrical power measurement system, and an electrical matching circuit (Figure 1A). Ultrasound parameters were a 25 ms pulse duration, 1 Hz pulse repetition frequency, 1 MHz US frequency, and 60 s treatment duration. Acoustic pressures between 0.1 MPa and 0.5 MPa were investigated. Before sonication, the animals were anesthetized by the inhalation of isoflurane (5% induction and 2% maintenance, Axience, Pantin, France). Then, a bolus of 0.1 mL sulfur hexafluoride microbubbles (8 µL/mL, SonoVue, Bracco Imaging, Milan, Italy) was injected intravenously via the dorsal penis vein. The choice of penis vein as the injection site was motivated by its reproducibility, as shown in previous publications [13]. Sonication was performed immediately after. The other eye was used as an internal control.

### 2.3. Fundus Photography, Angiography and Optical Coherence Tomography (OCT) Imaging

Pupils were dilated with one drop of 0.5% tropicamide (Mydriaticum, Théa, Clermont-Ferrand, France) and one drop of 5% phenylephrine (Neosynephrine, Europhta, Monaco). Fundus images and angiographies were taken on a Micron IV machine (Phoenix Technology Group, Pleasanton, CA, USA) after intra-peritoneal injection of 0.1 mL of 1% fluorescein or intraperitoneal injection of 0.15 mL of 2 mmol/L fluorescein isothiocyanate (FITC) marked-dextrans (10 kDa, 40 kDa, and 150 kDa, Sigma-Aldrich, FD10S, FD40S, and FD150S). OCT imaging was performed on a Bioptigen OCT system (Leica microsystems, Durham, NC, USA). Eyes were kept moisturized with a drop of carbomer 980 (Lubrithal, Dechra, Northwich, UK) during the whole procedure.

### 2.4. Fundus Electroretinography (ERG)

Mice were kept in the dark overnight and anesthetized using a mixture of ketamine (80 mg/kg, Axience, Pantin, France) and xylazine (8 mg/kg, Rompun 2%, Bayer HealthCare, France). Their pupils were dilated with one drop of 0.5% tropicamide and the cornea was anesthetized by the local application of one drop of oxybuprocaine (1.6 mg/0.4 mL, Théa, Clermont-Ferrand, France). A small wire loop electrode contacting the cornea through a layer of Lubrithal was used to record the retinal response, with needle electrodes placed in the cheeks and back used as the reference and ground electrodes, respectively. Body temperature was maintained at ~37 °C with a heating pad. The light stimulus was provided by a white LED in a Ganzfeld stimulator (ColorDome Lab Cradle, Diagnosys LLC). Dark-adapted responses were measured in darkness during flash stimulation (0.003, 0.03, 0.3, 3, and 10 cd.s/m^2^). Photopic cone ERGs were recorded in response to a flash (10 cd.s/m^2^) on a rod-suppressing background (20 cd.s/m^2^) after five minutes of light adaptation. Responses were amplified and filtered (1 Hz-low and 300 Hz-high cutoff filters) with a one-channel DC-/AC-amplifier. Each dark-adapted or photopic ERG response was the mean of five responses from a set of five stimulatory flashes. Flicker ERGs were recorded at 10 Hz. The left eye was used as an internal control for the sonicated right eye.

### 2.5. Immunohistochemistry

Mice were euthanized and eyes were enucleated, fixed for 1 h in 4% paraformaldehyde, then rinsed and sectioned at the limbus. The cornea and lens were discarded. Eyecups containing the retina, retinal pigment epithelium, choroid, and sclera were surgically separated from the vitreous and incubated with anti-ZO1 (1:200, Merck Millipore) or anti-isolectin GS-IB4 antibodies (Alexa fluor 568 coupled, 1:100, ThermoFisher Scientific, Waltham, MA, USA) at 4 °C. After washing, sections were incubated for 2 h with an Alexa Fluor 488-conjugated donkey anti-rat IgG (1:500, ThermoFisher Scientific) and counterstained with Hoechst (1:1000, ThermoFisher Scientific). Eyecups were then washed, flat mounted, and fluorescent staining signals were captured with an Olympus FV1000 confocal microscope equipped with 405, 488, 543, and 633 nm lasers. ZO-1 border staining was quantified by a ranking score system described by Muthusamy et al. [14] with a scale ranging from 1 to 5 as follows: 1—near complete loss of border staining (staining up to 25%); 2—25% to 50% continuous border staining; 3—50% to 75% continuous border staining; 4—75% to 100% continuous border staining; 5—complete continuous border staining. Scoring was completed in a masked fashion by two independent observers.

### 2.6. Reverse Transcription and Quantitative Polymerase Chain Reaction

Reverse transcription quantitative polymerase chain reaction (RT-qPCR) was used to measure the mRNA expression levels. The total RNA was extracted from the retinas, RPE, and choroids using the Nucleospin RNA (Macherey-Nagel, Düren, Germany Cat. #740902) according to the manufacturer’s instructions and converted to cDNA using the QuantiTect Reverse Transcription Kit (Qiagen, Hilden, Germany Cat. #205314). Each reverse transcription assay was performed in a 20 μL reaction. Subsequent real-time qPCR was performed using cDNA and SYBR Green PCR Master Mix (ThermoFisher Scientific, Cat. #4367659) in a StepOne Plus Real-Time PCR system (ThermoFisher Scientific, Applied Biosystems™) with the following profile: 10 min at 95 °C, followed by a total of 40 two-temperature cycles (15 s at 95 °C and 1 min at 60 °C). To verify the purity of the products, a melting curve was produced after each run according to the manufacturer’s instructions. Results were expressed as fold induction after normalization by *Rps26* gene expression. Primers for RT-qPCR were purchased from Integrated DNA Technologies^®^ (Coralville, Iowa, IA, USA) to study the expression of the following genes: retina/RPE function and homeostasis: 11-cis retinol dehydrogenase 5 (*Rdh5*), retinal pigment epithelium-specific 65 kDa protein (*Rpe65*), orthodenticle homeobox 2 (*Otx2*), and glial fibrillary acidic protein (*Gfap*); tight and adherens cell junctions: zonula occludens (*Zo*)-1, occludin (*Ocl*), cadherin 5 (*Cdh5*), and Claudin 5 (*Cldn5*); inflammation: chemokine (C–C motif) ligand 2 (*Ccl2*), interleukin 6 (*Il6*), tumor necrosis factor alpha (*Tnfa*); apoptosis: caspase 9 (*Cas9*), B cell leukemia/lymphoma 2 (*Bcl2*), and BCL2-associated X protein (*Bax*) (sequences upon request).

### 2.7. Statistical Analysis

GraphPad Prism 9.5.1 (GraphPad Software) was used for the data analysis and graphic representation. All values are reported as mean ± SEM. Statistical analysis was performed by Mann–Whitney or ANOVA for comparison among means depending on the experimental design. The *p*-values are indicated in the figure legends.

## 3. Results

### 3.1. Determination of the Optimal Ultrasound Acoustic Pressure

Several pressure parameters were tested between 0.10 and 0.50 MPa. Figure 1 shows the results of a typical experiment with all of the tested US pressures. In the absence of microbubbles, no effect of sonication was visible on the fundus pictures (Figure 1B), fluorescein angiography (Figure 1C), or OCT (Figure 1D) at any of the tested acoustic pressures. We then performed microbubble-assisted sonication. With microbubbles at 0.10 MPa, no effect of sonication was visible on the fundus photography, and inconsistent leakage was observed on fluorescein angiography (Figure 1E,F). At 0.15 MPa, while no alteration was visible on the fundus photography, leakage was consistently present on the angiography (Figure 1F). In contrast, retinal hemorrhages and cotton-wool spots were found as signs of leakage and ischemia with 0.25 MPa, and to a higher extent at 0.50 MPa. In this experimental condition, fluorescein angiography also showed severe leakage except in ischemic areas (Figure 1F). On OCT, no change was visible for the 0.10 and 0.15 MPa acoustic pressures compared to the control (Figure 1G), whereas retinal edema and subretinal detachment were seen as manifestations of increased fluid leakage with the 0.50 MPa acoustic pressure (Figure 1G, white arrows). Increased hyperreflectivity of the internal retinal layers was also observed as a sign of associated ischemia in the 0.25 MPa and 0.50 MPa groups (Figure 1G, white asterisks).

Immunohistological examination was performed on sonicated and unsonicated eyes. The integrity of the retinal vessel tight junctions was assessed using ZO-1 staining (Supplementary Figure S1A,B), showing no visible change at 0.10 and 0.15 MPa. On the other hand, the staining seemed less regular in some of the 0.50 MPa conditions, showing a possible alteration of the BRB. However, when using a semi-quantitative quantification of ZO-1 staining, we did not find any statistical difference between the acoustic pressures (Supplementary Figure S1D). Using isolectin GS-IB4 (IB4) staining (Supplementary Figure S1C), no change in the vascular architecture was seen with any of the acoustic pressures. However, we noticed the presence of extravascular IB4-positive cells in the 0.25 and 0.50 MPa groups, which has been shown to be typical of activated monocytes/macrophages [15], suggesting their possible extravasation or recruitment at the site of sonication.

### 3.2. Angiographic Leakage Quantification

**Figure 2:**
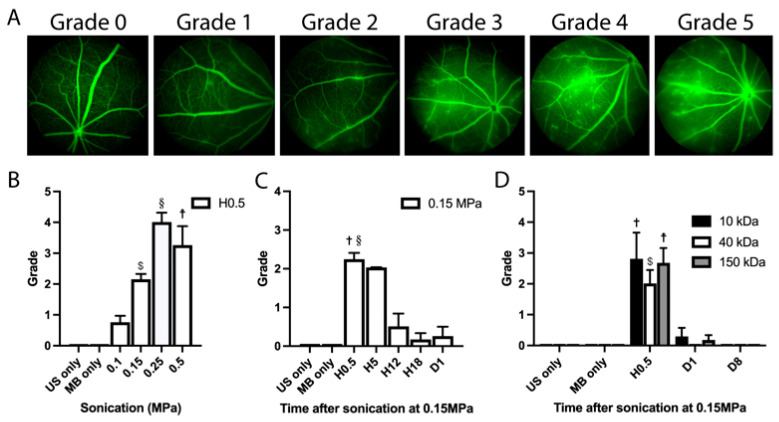
(**A**) Angiography grading scheme used for leakage assessment: grade 0 corresponds to the absence of leakage; grade 1 to the presence of a mild leakage in 1 or 2 quadrants but not in the posterior pole; grade 2 to the presence of numerous leakages in one or 2 quadrants but not in the posterior pole; grade 3 to the presence of a mild and diffuse leakage in the posterior pole; grade 4 to the presence of a major leakage in the posterior pole but without optic disc involvement; grade 5 to the presence of a major leakage in the posterior pole including in the optic disc. (**B**) Quantification of fluorescein leakage according to the grading described in (**A**), 30 min after sonication (Hour(H)0.5) with different acoustic pressures. One-way ANOVA for multiple comparisons: 0.15 MPa vs. MB only, $ *p* < 0.0001; 0.25 MPa vs. MB only, § *p* < 0.0001; 0.50 MPa vs. MB only, ☨ *p* = 0.0001; 0.10 MPa vs. MB only, not significantly different. (**C**) Quantification of fluorescein leakage according to the grading described in (**A**) at H0.5, H5, H12, H18, and Day (D)1 of sonication at 0.15 MPa. One-way ANOVA for multiple comparisons: H0.5 vs. MB only, † *p* < 0.0001; H0.5 vs. H12, § *p* < 0.01; H0.5 vs. H5: not significantly different. (**D**) Leakage quantification after intraperitoneal injection of 2 mM of 10, 40 and 150 kDa FITC-marked dextrans and sonication at 0.15 MPa, at H0.5, D1 and D8 of sonication. (n = 5–10/group). One-way ANOVA for multiple comparisons: 10 kDa H0.5 vs. MB only, † *p* < 0.001; 40 kDa H0.5 vs. MB only, $ *p* < 0.0001; 150 kDa H0.5 vs. MB only, ☨ *p* < 0.0001; all dextrans D1 and D8 vs. MB only, not significantly different. US: ultrasound; MB: microbubbles.

To quantify the degree of BRB opening following sonication in the absence of a commonly admitted classification, we designed an angiographic vessel leakage grading scheme. This grading scale shares similarities with the one used for uveitis in mice [16]. Angiographic leakage was graded from 0 to 5 (Figure 2A) depending on the site and intensity of leakage: grade 0 corresponded to the absence of leakage; grade 1 to the presence of a mild leakage in 1 or 2 quadrants, but not in the posterior pole; grade 2 to the presence of numerous leakages in one quadrant but not in the posterior pole; grade 3 to the presence of a mild diffuse leakage in the posterior pole; grade 4 to the presence of a major leakage in the posterior pole but without optic disc involvement; grade 5 to the presence of a major leakage in the posterior pole including in the optic disc.

We graded the degree of fluorescein leakage in sonicated and unsonicated eyes (Figure 2B). No leakage was observed in any of the control eyes (US only or microbubbles (MB) only). Thirty minutes (Hour (H)0.5) after sonication, we observed a mean leakage grade of 0.75 +/− 0.75 SD with the 0.1 MPa acoustic pressure, 2.39 +/− 1.39 SD with 0.15 MPa, 4.00 +/− 0.71 SD with 0.25 MPa, and 3.25 +/− 1.26 SD with 0.5 MPa. Since the 0.15 MPa acoustic pressure was associated with significant fluorescein leakage but no visible tissue alteration on the fundus or OCT imaging (Figure 1), we investigated the duration of the BRB opening in this experimental condition. In Figure 2C, we showed that the degree of fluorescein leakage was stable between H0.5 and H5 (mean leakage grade of 2.14 +/− 1.37 SD and 2.00 +/− 0 SD respectively at H0.5 and H5, *p* = 0.99), decreasing to 0.50 +/− 0.84 at H12 (*p* = 0.99 versus control), 0.17 +/− 0.41 at H18 (*p* = 0.99 versus control) and vanishing almost completely by Day(D)1 (mean leakage grade of 0.25 +/− 0.50 SD at D1, *p* = 0.99 versus control). Therefore, the 0.15 MPa condition was selected as the most optimal acoustic pressure for the following experiments. Regarding the duration of sonication, we investigated the effects of durations ranging from 60 to 120 s, showing that a 60-s treatment protocol was sufficient to induce a BRB opening (Supplementary Figure S2A,B).

In order to determine the size of the leaked molecules through the internal BRB with the 0.15 MPa acoustic pressure, the same leakage grading was performed after the injection of fluorescent dextrans of different molecular weights (Figure 2D). No leakage was observed in any of the control eyes. At H0.5, we observed a mean leakage grade of 2.8 +/− 1.92 SD with the 10 kDa, 2.0 +/− 1.0 SD with the 40 kDa, and 2.7 +/− 1.21 with the 150 kDa dextran, being statistically significant from the control for all dextran sizes (*p* < 0.001, *p* < 0.0001, and *p* < 0.0001, respectively). At day 1, the mean grade was 0.26 +/− 0.76 SD with the 10 kDa, 0 +/− 0 SD with the 40 kDa, and 0.17 +/− 0.41 for the 150 kDa dextran (*p* = 0.93 for the 10 kDa, *p* = 0.99 for the 40 kDa, and *p* = 0.96 for the 150 kDa dextran compared to the control). At day 8, no leakage was observed with any of the dextrans.

### 3.3. Safety Analysis

**Figure 3:**
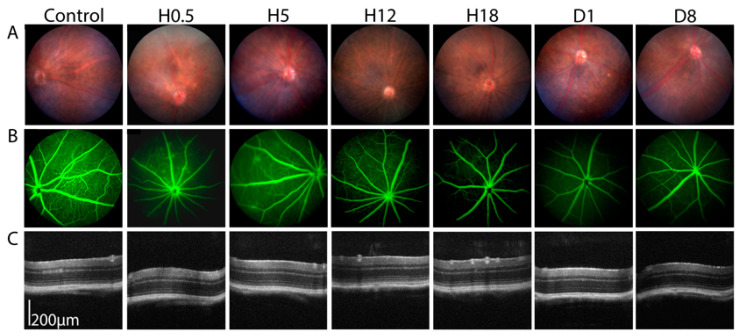
Sonication at an acoustic pressure of 0.15 MPa. (**A**) Fundus retinophotographs at Hour (H)0.5, H5, H12, H18, Day (D) 1 and 8 after sonication, field of view 50°. (**B**) Fluorescein angiography H0.5, H5, H12, H18, D1, and D8 after sonication, field of view 50°. (**C**) Optical coherence tomography at H0.5, H5, H12, H18, D1, and D8 after sonication.

After choosing the 0.15 MPa condition as the most suitable acoustic pressure to transiently open the BRB, we assessed the safety at different timepoints after sonication. At H0.5, H5, H12, H18, day 1, and day 8 of monitoring, we observed no anatomical change on the fundus photographs or OCT imaging (Figure 3A,C). On the fluorescein angiography, while leakage was seen until H5, it resolved almost completely after 12 h (Figure 3B).

**Figure 4:**
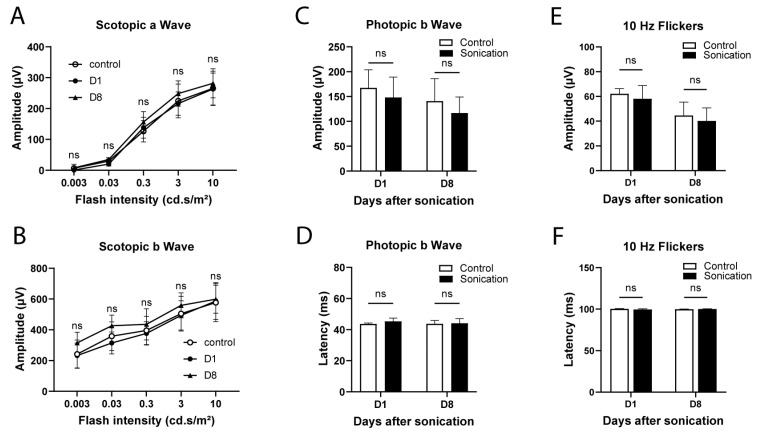
Electroretinography at Day (D)1 and 8 of sonication in the sonicated (0.15 MPa) and control (contralateral) eyes. (**A**) Scotopic a-wave amplitude. (**B**) Scotopic b-wave amplitude. Photopic b-wave amplitude (**C**) and latency (**D**). The 10 Hz flicker amplitude (**E**) and latency (**F**). (n = 3–8/group), two-way ANOVA (**A**,**B**) or Mann–Whitney (**C**–**F**) comparison versus the controls, ns: not statistically significant.

Electroretinography (ERG) was performed in order to better evaluate the safety in terms of visual function (Figure 4). Mice were tested in both scotopic (a- and b-waves) and photopic conditions (amplitude and latency of b-wave; 10 Hz flickers), as previously described. In scotopic conditions (Figure 4A,B) and irrespective of the flash intensity, there was no significant difference between the sonicated and control eyes regarding a-wave (*p* = 0.26 and b-wave amplitudes (*p* = 0.32). In photopic conditions (Figure 4C,D), the ERG waveforms in response to a 10 cd.s/m^2^ flash light and 10 Hz flickers (Figure 4E,F) showed no difference between the sonicated and control eyes.

To understand the effects and safety of sonication at 0.15 MPa on gene expression, we performed quantitative RT-PCR from the retinas and RPE/choroids extracted from the sonicated and control eyes at different timepoints. We analyzed the expression of key genes involved in different pathways including normal cellular functions, cell junctions, inflammation, and apoptosis.

**Figure 5:**
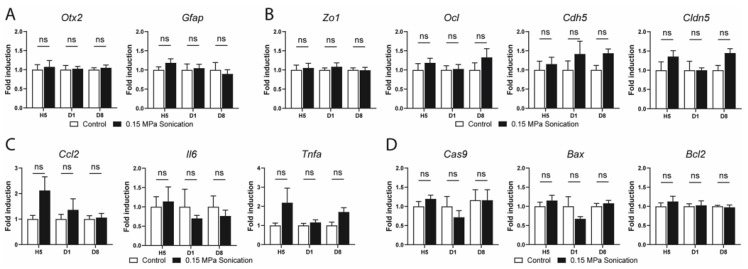
RT-qPCR in retinal extracts of sonicated and non-sonicated (contralateral) control eyes at hour(H) 5, day(D) 1 and 8 after sonication at 0.15 MPa. Relative expression of (**A**) genes implicated in retinal homeostasis: *Otx2* and *Gfap*; (**B**) genes implicated in tight and adherens junctions: Zo1, *Ocl*, *Cdh5*, and *Cldn5*; (**C**) inflammation genes: *Ccl2*, *Il6*, and *Tnfa*; (**D**) genes implicated in apoptosis: *Cas9*, *Bax*, and *Bcl2*. Gene expression was calculated relative to *Rps26* expression. (n = 5–6/group, Mann–Whitney, comparison versus the control, ns: not statistically significant); *Rdh5*: 11-cis retinol dehydrogenase 5; *Rpe65*: retinal pigment epithelium-specific 65 kDa protein; *Otx2*: orthodenticle homeobox 2; *Gfap*: glial fibrillary acidic protein; *Ocl*: occludin; *Cdh5*: cadherin 5; *Cldn5:* claudin 5; *Ccl2*: chemokine (C–C motif) ligand 2; *Il6*: interleukin 6; *Tnfa*: tumor necrosis factor alpha; *Cas9*: caspase 9; *Bcl2*: B cell leukemia/lymphoma 2; *Bax*: BCL2-associated X protein.

**Figure 6:**
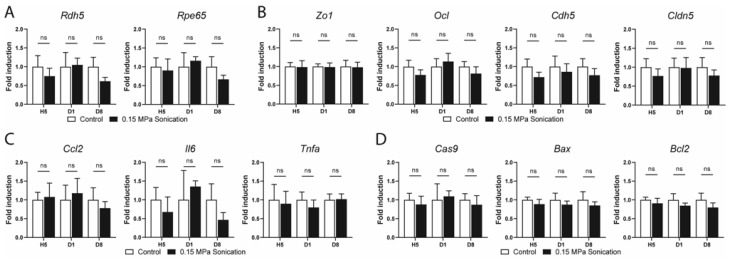
RT-qPCR in the RPE/choroid extracts of the sonicated and non-sonicated (contralateral) control eyes at Hour (H)5, Day (D)1, and 8 after sonication at 0.15 MPa. (**A**) RPE functions: *Rdh5* and *Rpe65*, (**B**) genes implicated in the tight and adherens junctions: *Zo1*, *Ocl*, *Cdh5*, and *Cldn5*, (**C**) inflammation-related genes: *Ccl2*, *Il6*, and *Tnfa*, and (**D**) genes implicated in apoptosis: *Cas9*, *Bax*, and *Bcl2*. Gene expression was calculated relative to *Rps26* expression. (n = 5–6/group, Mann–Whitney, comparison versus control, ns: not statistically significant). *Rdh5*: 11-cis retinol dehydrogenase 5; *Rpe65*: retinal pigment epithelium-specific 65 kDa protein; *Otx2*: orthodenticle homeobox 2; *Gfap*: glial fibrillary acidic protein; *Ocl*: occludin; *Cdh5*: cadherin 5; *Cldn5*: claudin 5; *Ccl2*: chemokine (C-C motif) ligand 2; *Il6*: interleukin 6; *Tnfa*: tumor necrosis factor alpha; *Cas9*: caspase 9; *Bcl2*: B cell leukemia/lymphoma 2; *Bax*: BCL2-associated X protein.

In the mature retina, *Otx2* is expressed in bipolar cells and photoreceptors [17], while *Gfap* is preferentially expressed in astrocytes [18] and activated Müller cells [19]. No change in the expression of these two genes was observed up to day 8 in the sonicated retinas when compared to the internal controls (Figure 5A). RDH5 and RPE65 are two key proteins in the visual cycle in RPE cells [20,21]. Neither gene showed any modification in their respective expression in the RPE/choroid samples of the sonicated versus control eyes (Figure 6A). The expression of genes involved in cell junctions (tight junctions: *Zo1*, *Ocl*, *Cldn5*; and adherens junctions: *Cdh5*) was investigated at different timepoints, showing no difference between the sonicated and unsonicated eyes in the retina (Figure 5B) or RPE/choroid extracts (Figure 6B). The expression of genes involved in the inflammation pathways (*Ccl2*, *Il6* and *Tnfa*) was not statistically different between the sonicated and unsonicated eyes at all timepoints in the retinas (Figure 5C) and RPE/choroid samples (Figure 6C). Finally, genes involved in apoptosis, namely, *Cas9*, *Bax*, and *Bcl2* showed a comparable expression to the same-day controls in the retinas (Figure 5D) and RPE/choroids (Figure 6D).

In order to show the toxic effects of sonication at higher acoustic pressures, we performed the same quantitative RT-PCR experiments on the retinas and RPE/choroid extracts from eyes sonicated at 0.25 MPa (Supplementary Figure S3). We showed that sonication at such an acoustic pressure was toxic to the retina, as shown by the increased expression of *Gfap* and inflammation-related genes (*Ccl2*, *Il6* and *Tnfa*). In the RPE/choroid extracts, the expression of *Ccl2* was also increased, while that of *Il6* showed a trend toward an increase.

## 4. Discussion

This study is a proof-of-concept that the BRB can be efficiently, transiently, and safely opened using a 1 MHz unfocused US sonication coupled with microbubbles. In this study, we showed that an optimized US acoustic pressure of 0.15 MPa allowed for a safe and transient inner BRB opening in mice, allowing macromolecules up to 150 kDa to reach the retina, which could be of interest in posterior segment diseases requiring systemic treatments with drugs known to bare a limited retinal penetration. The development of such a technology would also be beneficial in terms of decreasing the burden of intravitreal injections in these diseases.

### 4.1. Optimization of Sonication Parameters

In this study, we first aimed at finding the most appropriate sonication parameters that would not only allow the BRB to be efficiently opened, but also secure a transient and safe opening while limiting the number of animals to be sacrificed. In fact, various parameters can be tailored during a sonication protocol including the acoustic pressure, pulse duration, pulse repetition frequency, US frequency, and treatment duration. We focused on tailoring the acoustic pressure and treatment duration while keeping all other parameters unchanged, choosing both a fixed pulse duration and frequency, matching previous studies using the same device on the blood–brain barrier (BBB) [12,22,23]. Regarding the duration of sonication, we investigated the effects of durations ranging from 60 to 120 s, showing eventually that a 60-s treatment protocol was sufficient to induce a BRB opening (Supplementary Figure S2). This 60-s duration was shorter than what would be necessary to open the BBB [12,22,24], but similar to what Park et al. [9] previously published.

In contrast to Park et al., we found that peak negative pressures ranging from 0.15 to 0.5 MPa were enough to open the BRB, while they found figures that were twice as high (0.78 to 1.06 MPa) to open the BBB. This could be due to the fact that they used a lower US frequency (690 kHz), a shorter burst duration, and a different US contrast agent, or to a constitutional difference between the mouse that we used and the rat models that Park et al. used. It should be noted that the efficacy of sonication can be dependent on anesthetic protocols, as Montero and colleagues showed a negative effect of anesthesia using inhaled isoflurane and oxygen on the quality of the BBB opening [24]. In fact, isoflurane and oxygen are known to decrease the effect of microbubbles on the BBB via vasoactive effects (including vasoconstriction, vasodilation and blood flow) and decreased microbubble circulation times [25].

Regarding the acoustic pressure optimization experiments, we observed a mild and inconsistent BRB opening using 0.10 MPa, allowing us to dismiss this acoustic pressure. On the other hand, replicable leakage of fluorescein was consistently found with acoustic pressures ranging from 0.15 to 0.5 MPa. However, the 0.5 MPa sonication was systematically associated with overt signs of leakage and ischemia on the fundus photographs (hemorrhages, cotton wool spots, retinal whitening, etc.) and OCT (increased retinal thickness, subretinal fluid, increased reflectivity of internal retinal layers), showing poor clinical tolerance. Moreover, the 0.50 MPa acoustic pressure seemed to be responsible for an at least transient architectural alteration of tight junctions, seen as an irregular flat mount staining of the retinal vessels using anti ZO-1 antibodies. However, this trend was not confirmed when semi-quantitative quantification of ZO-1 staining was performed. The 0.50 MPa sonication also seemed to trigger an extravasation or recruitment of what could be mononuclear phagocytes [15], demonstrated via an isolectin GS-IB4 staining of flat mounted retinas. These toxic observations were also visible (although to a lower degree) with an acoustic pressure of 0.25 MPa, suggesting that this threshold should not be exceeded, as it has been shown that mononuclear phagocytes trigger toxic reactions that can be harmful for retinal function [26].

### 4.2. Duration of BRB Disruption, Efficacy of Sonication and the Size of Leaked Molecules

Microbubble-associated sonication enhances drug delivery via two mechanisms of action: the first being the direct cell proximity of microbubbles to endothelial cells, resulting in a stimulated endocytosis or pore formation (so called sonoporation), depending on the acoustic pressure [27,28]; the second being an increased vascular permeability as a result of microbubble cavitation, resulting in a disruption of tight junctions (sometimes called sonopermeation, the term also used to encompass all of the aforementioned mechanisms [29]). While the absence of confirmed ZO-1 alterations may suggest a mechanism of sonoporation, we can still postulate that a transient permeabilization of tight junctions may also play a role in our observations, as we reported fluorescein leakage being still present after 5 to 6 h. This duration is consistent with previous studies showing the recovery of tight junctions around 9 h after sonication [30]

In the absence of a consensual classification, the degree of leakage was assessed qualitatively using a grading scheme that we developed based on fluorescein angiography imaging, as described in the methods section. At H0.5, we showed that the leakage of fluorescein (376 Da) using the 0.15 MPa acoustic pressure was consistently seen in all of the tested mice. The leakage disappeared almost completely from H12. In order to assess the size of the leaked molecules, we performed angiography using FITC-marked dextrans of different sizes (10–150 kDa) and at different timepoints (Figure 2D). We showed that all dextrans leaked at H0.5, and that the leakage disappeared almost completely by day 1, being completely absent at day 8. This demonstrates that BRB opening can theoretically allow the extravasation of molecules up to 150 KDa in size. To confirm this, immunohistochemistry using FITC-marked anti-anti-mouse immunoglobulin (Ig)G (of size up to 150 kDa) antibodies also showed the presence of leaked antibodies in the retina at H0.5 (Supplementary Figure S1E). Furthermore, the 150 kDa FITC-dextrans were also visible in the retina using confocal microscopy on flat mounts (Supplementary Figure S1F).

### 4.3. Safety

To our knowledge, this was the first study to assess the anatomical and functional safety of microbubble-associated sonication using the optimized acoustic pressure of 0.15 MPa. In terms of anatomy, the fundus and OCT exams showed no modification of the retinal, RPE, or choroidal architecture up to 8 days of sonication. Immunostaining of tight junctions using anti-ZO1 antibodies did not show any noticeable modification of the retinal vessel endothelial cells or RPE cell architecture (Supplementary Figure S4). Staining with isolectin was also unremarkable and did not show any vessel alteration nor extravasation or the recruitment of potential inflammatory cells in the retina.

In terms of visual function, we performed electrophysiological tests on the day after sonication by using full-field and flicker ERGs providing an insight into the physiology and integrity of the retina [31] by discriminating the differential functions of photoreceptors (rods and cones) and bipolar cells. We did not observe any difference in the scotopic or photopic responses compared to the control contralateral eyes, supporting the functional safety of sonication at 0.15 MPa.

We also explored the possibility of cellular dysregulations through gene expression analysis in the retinal and RPE/choroid extracts. Regarding the cell junction gene expression, we did not observe any change up to 8 days after sonication in genes specific to tight and adherens junctions. Regarding the retina and RPE specific genes, we did not observe any change in the expression of *Otx2*, *Gfap*, *Rdh5*, or *Rpe65*. Sonication can theoretically trigger inflammatory responses and the activation of microglial cells, as shown by Ahmed et al. [32] in the brain. We therefore investigated the expression of inflammation genes including *Ccl2*, *Il6*, and *Tnfa*. Despite the presence of a slight trend toward an increased expression of *Ccl2* and *Tnfa* in the sonicated mouse retinas, such an increase was only seen at H5 (without being statistically significant from the baseline) and disappeared completely at days 1 and 8. No other statistically significant differential expression of inflammation genes was seen in the retinas and RPE/choroids of the sonicated versus control eyes at any of the investigated timepoints. On the other hand, with the 0.25 MPa pressure, we observed a significant increase in the expression of *Gfap*, *Il6*, *Ccl2*, and *Tnfa* in the mouse retinas, and an increased expression of *Ccl2* in the RPE/choroids at day 1, showing that this threshold pressure induces an inflammatory response that had not been seen with the 0.15 MPa acoustic pressure (Supplementary Figure S3). Finally, genes implicated negatively or positively in the pathway of apoptosis were investigated, showing no statistically significant difference between the sonicated and control eyes, suggesting no increase in cellular mortality.

### 4.4. Perspectives

The most developed and promising application of sonopermeation is its current use as an enhancer of BBB opening. Sonication has in fact been successfully used in the treatment of brain tumors: following pre-clinical experimentation in rabbits [22], mice [23], and primates [33], clinical trials have shown an improved efficacity of systemic chemotherapy associated with microbubble-assisted sonication in recurrent cerebral glioblastoma [6]. In ophthalmology, one logical potential application of transient BRB opening would be to enhance the efficacy of systemic chemotherapy in ocular cancers. Retinoblastoma (Rb) is a rare but life-threating cancer affecting children. In most cases, treatment is at least partly based on systemic chemotherapy [34]. The systemic chemotherapies that are currently used in Rb include carboplatin, vincristine, etoposide, topotecan, and melphalan [34] with the respective molecular weights of 371, 588, 824, and 305 Da. These chemotherapies do not necessarily efficiently cross the BRB [35], providing a justification for the use of microbubble-associated sonication. To our knowledge, there has been no study investigating the role of systemic chemotherapy enhanced by the local use of microbubble-assisted sonication in animal models of retinoblastoma. Our preliminary study provides a useful background for future studies on this subject.

In summary, microbubble-associated sonication could represent a transient and safe way to open the blood–retinal barrier and hence improve retinal drug delivery.

## Data Availability

Data is not available due to privacy restrictions.

## Supplementary Materials

The following supporting information can be downloaded at: https://www.mdpi.com/article/10.3390/pharmaceutics15071896/s1, Figure S1. Immunohistochemistry in retinas; Figure S2. Multimodal imaging of C57/BL6J male mice undergoing low-intensity pulsed ultrasound during 60 or 120 s at 0.15 MPa; Figure S3. RT-qPCR in the retinal and RPE/choroid extracts of the sonicated and unsonicated eyes at Day (D)1 after sonication at 0.25 MPa; Figure S4. Confocal microscopy of ZO-1 (zonula occludens) immunostaining depicting vascular tight junctions on retinal pigment epithelium/choroid flat mounts.
