# Supplementary material for: Efficacy and Safety of Low-Intensity Pulsed Ultrasound-Induced Blood–Retinal Barrier Opening in Mice
Source: Pharmaceutics. 2023 Jul 6;15(7):1896. doi: 10.3390/pharmaceutics15071896 (PMC10384184; doi:10.3390/pharmaceutics15071896)
Supplement: Supplementary file 1 [file pharmaceutics-15-01896-s001.zip › pharmaceutics-2445399-supplementary.pdf]

pharmaceutics

MDPI

Article

Efficacy and Safety of Low-Intensity Pulsed Ultrasound-Induced Blood–Retinal Barrier Opening in Mice

Alexandre Bourdin [1,†], Manon Ortoli [1,†], Remi Karadayi [1], Lauriane Przegralek [1], Florian Sennlaub [1], Bahram Bodaghi [2], Xavier Guillonneau [1], Alexandre Carpentier [3,4,5,‡] and Sara Touhami [1,2,*,‡]

Supplementary Material

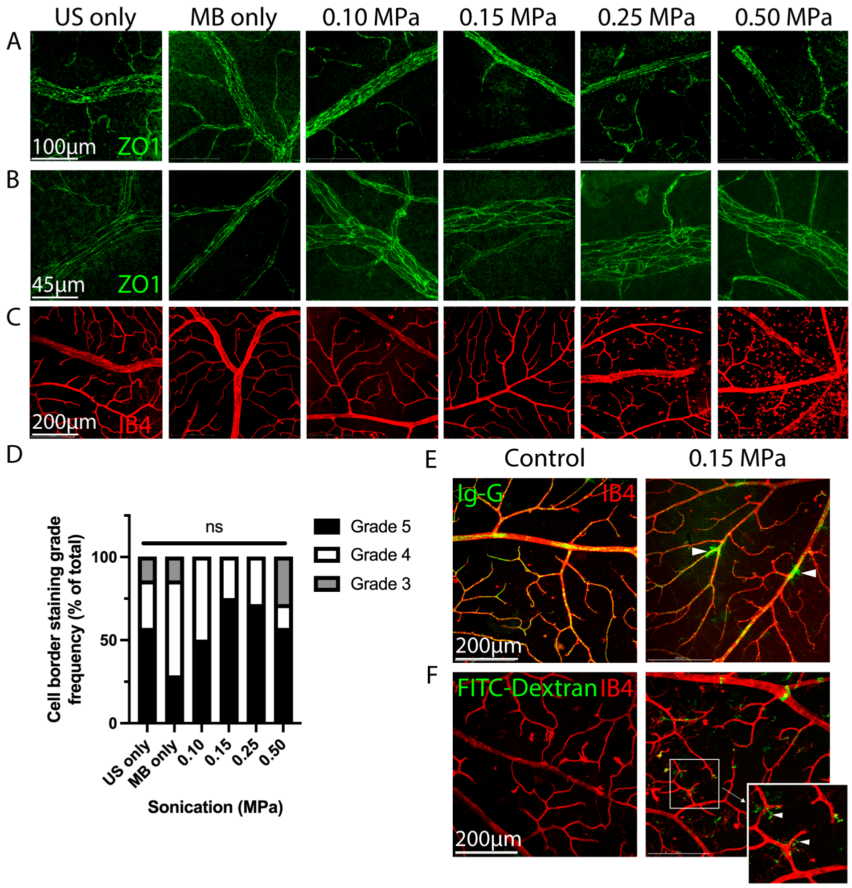

Supplementary Figure S1. Confocal microscopy of (**A**) ZO-1 (Zonula Occludens) immunostaining depicting vascular tight junctions on retinal flat mounts, (**B**) High magnification of the ZO-1 immunostaining and (**C**) Isolectin GS-IB4 immunostaining depicting retinal vessel architecture and presence of mononuclear phagocytes, after microbubble-assisted sonication using different acoustic pressures in C57/BL6J male mice. (**D**) Quantification of the ZO1 signal shown in (**B**) using Muthusamy et al. technique (see Materials and Methods), Fisher's exact test (n = 8 images/group). (**E**) Confocal microscopy of Immunoglobulin(Ig) G (green) and Isolectin GS-IB4 immunostaining (red) in C57/BL6J male mice showing extravasation (white arrow heads) of IgGs in eyes undergoing microbubble-assisted sonication at 0.15MPa versus controls. (n = 5-8/group). (**F**) Confocal microscopy of 150 kDa FITC-dextrans (green) injected before sonication showing extravasation at 0.15 MPa versus controls. The white square depicts a magnified region showing better the extravasation process (white arrowheads).

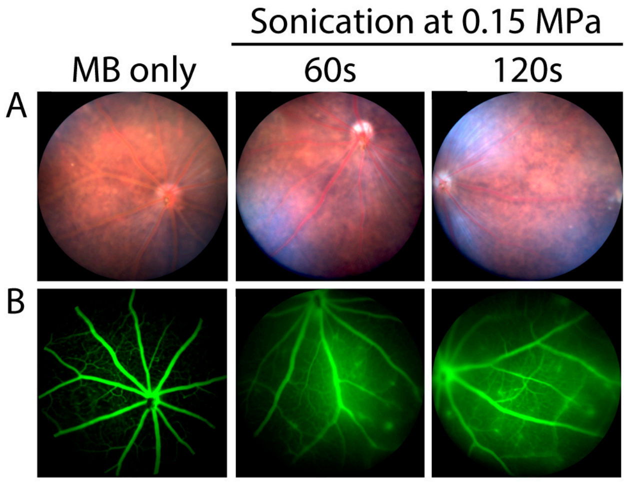

Supplementary Figure S2. Multimodal imaging of C57/BL6J male mice undergoing low-intensity pulsed ultrasound during 60 or 120 seconds at 0.15 MPa. Fundus retinophotograph, field of view 50°. (**A**) Fluorescein angiography (5min, late phase), field of view 50° (**B**). MB: microbubbles.

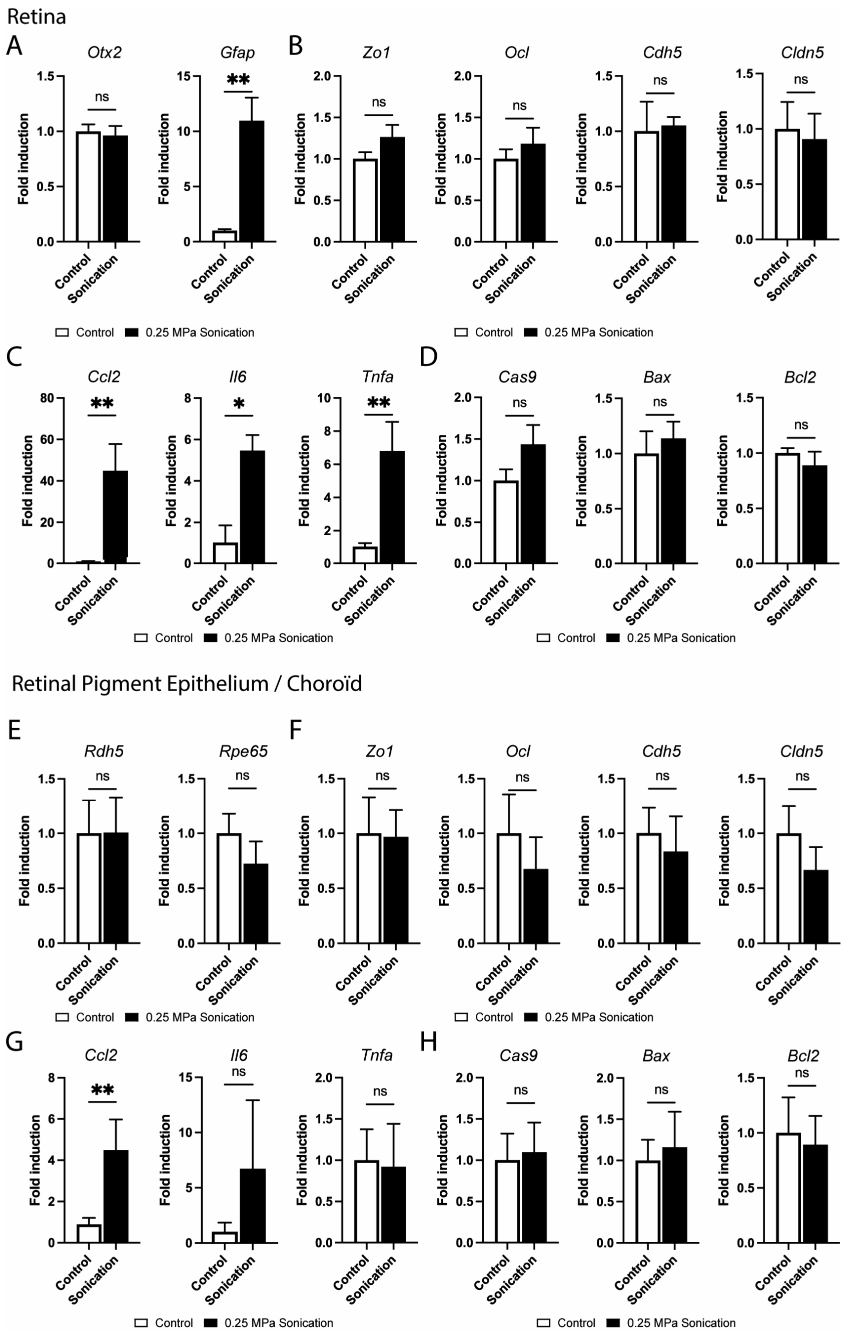

Supplementary Figure S3. RT-qPCR in retinal and RPE/choroid extracts of sonicated and unsonicated (contralateral) eyes at day(D) 1 after sonication at 0.25MPa. Relative expression of: (**A** retina and **E** RPE/choroids) genes implicated in tissue homeostasis: Otx2, Gfap, Rdh5, Rpe65, (**B** retina and **F** RPE/choroid) genes implicated in tight and adherens junctions: Zo1, Ocl, Cdh5 and Cldn5, (**C** retina and **G** RPE/choroid) inflammation genes: Ccl2, Il6 and Tnfα, and (**D** retina and **H** RPE/choroids) genes implicated in apoptosis: Caspase 9, Bax and Bcl. Gene expression was calculated relative to Rps26 expression. (n = 5-6/group, Mann-Whitney, comparison versus control: $*P < 0.05$, $**P < 0.005$, ns: not statistically significant). RPE: retinal pigment epithelium. Rdh5: 11-cis retinol dehydrogenase 5. Rpe65: retinal pigment epithelium-specific 65 kDa protein. Otx2: orthodenticle homeobox 2. Gfap: glial fibrillary acidic protein. Ocl: occludin. Cdh5: cadherin 5. Cldn5: claudin 5. Ccl2: chemokine (C-C motif) ligand 2. Il6: interleukin 6. Tnfα: tumor necrosis factor alpha. Cas9: caspase 9. Bcl2: B cell leukemia/lymphoma 2. Bax: BCL2-associated X protein.

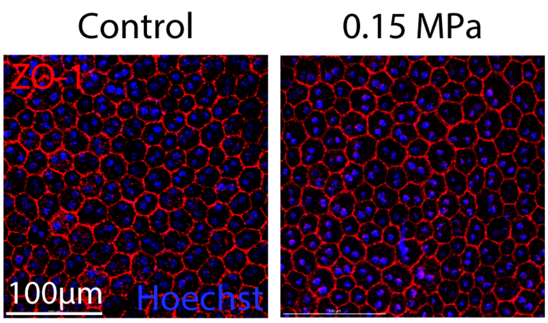

Supplementary Figure S4. Confocal microscopy of ZO-1 (Zonula Occludens) immunostaining depicting vascular tight junctions on retinal pigment epithelium/choroid flatmounts.
